# Optimizing CRISPR/Cas9 mutagenesis in *Drosophila* dendritic arborization neurons to avoid cytotoxicity

**DOI:** 10.1093/g3journal/jkag211

**Published:** 2026-09-03

**Authors:** Vinicius N Duarte, Viana Najafi, Katherine L Thompson-Peer

**Affiliations:** Department of Developmental and Cell Biology, University of California, Irvine, CA 92697, United States; Department of Developmental and Cell Biology, University of California, Irvine, CA 92697, United States; Department of Developmental and Cell Biology, University of California, Irvine, CA 92697, United States; Center for the Neurobiology of Learning and Memory, University of California, Irvine, CA 92697, United States; Sue and Bill Gross Stem Cell Research Center, University of California, Irvine, CA 92697, United States

**Keywords:** CRISPR, Cas9, mutagenesis, *Drosophila*, da neuron, sensory neuron, dendrites, cytotoxicity, dendrotoxicity

## Abstract

Genetic perturbations are one of the great strengths of the model organism *Drosophila melanogaster*, with approaches such as classical mutagenesis and RNA interference enabling a wealth of biological discoveries. A more recent approach for altering gene expression is CRISPR/Cas9-based mutagenesis. As with any new tool, however, its use must be optimized. High expression of Cas9 has been shown to cause cytotoxicity in some cell types, including class IV dendritic arborization (da) neurons. In this study, we provide evidence that Cas9 expression causes cytotoxicity in class I da neurons, in addition to class IV da neurons, both of which are widely used to study neuronal development and regeneration. We then systematically evaluated available Cas9 transgenes designed to titrate Cas9 expression, called uCas9 transgenes. We show that the expression of these uCas9 transgenes results in little to no cytotoxicity in various classes of da neurons. Immunostaining revealed drastic reductions in Cas9 protein levels for da neurons expressing the uCas9(L) transgene. Lastly, we demonstrate that the uCas9(L) transgene effectively and specifically gene edits in both class I and class IV da neurons, lowering the expression of GFP-tagged proteins and producing loss-of-function morphological phenotypes when targeting endogenous loci. Thus, we refine the use of CRISPR mutagenesis in *Drosophila* da neurons through titration of Gal4/UAS-mediated Cas9 expression using existing uCas9 transgenes, a feasible and flexible approach that may be useful for other labs encountering Cas9 cytotoxicity in their own model systems.

## Introduction

The CRISPR (clustered regularly interspaced short palindromic repeats) gene editing platform has revolutionized the research community's ability to probe gene function (Jinek et al. 2012). The use of CRISPR/Cas9 gene editing in *Drosophila melanogaster* has been established and iteratively improved upon by several groups, from its initial application in somatic and germline cells to the optimization of guide RNA constructs for increased mutagenesis efficiency (Bassett et al. 2013; Gratz et al. 2013; Kondo and Ueda 2013; Ren et al. 2013, 2014; Yu et al. 2013; Port et al. 2014; Port and Bullock 2016; Koreman et al. 2021). However, ectopic expression of the Cas9 protein, the endonuclease that induces DNA damage with specificity from guide RNA molecules, can cause cellular toxicity, as reported by multiple groups across a range of model systems (Jiang et al. 2014; Port et al. 2014, 2020; Xue et al. 2014; Yang et al. 2018). Interestingly, this may not always depend on endonuclease activity as expression of a nuclease-dead Cas9 protein resulted in cellular toxicity to the single-cell alga, Chlamydomonas reinhardtii (Jiang et al. 2014; Port et al. 2014). Thus, CRISPR/Cas9 mutagenesis must be refined when used in novel experimental contexts.

The dendritic arborization (da) neurons of *D. melanogaster* are a powerful system to interrogate neuronal development and regeneration (Grueber et al. 2002, 2003a, 2003b; Song et al. 2012). There are 4 different classes of da neurons (type I-IV) based on the increasing dendritic complexity. Class IV da neurons have highly branched dendritic arbors, and they continue growing throughout larval development. Due to the large and dynamic nature of the class IV dendrite arbor, class IV neurons are often the most sensitive to manipulations that affect dendrite development and outgrowth. Gal4/UAS-mediated expression of Cas9 protein in class IV sensory neurons results in severe cytotoxic reductions in dendrite arbor complexity, which can be prevented by generating enhancer-driven Cas9 constructs that lower Cas9 protein levels (Poe et al. 2019; Koreman et al. 2021). However, enhancer-driven Cas9 transgenes must be generated for each cell or tissue type and may still cause Cas9-associated defects depending on the strength of the enhancer and experimental contexts. Direct enhancer-driven Cas9 transgenes also cannot be combined with Gal80 temperature-sensitive regulation or GeneSwitch approaches for delayed Cas9 expression.

An alternative approach to titrating Cas9 expression levels may be achieved with transgenes containing upstream open reading frames (uORFs) of varying lengths that are inversely correlated with protein expression (Kozak 2001; Ferreira et al. 2013; Southall et al. 2013). This uORF strategy was applied to UAS-Cas9 transgenes in *Drosophila* (referred to as UAS-uCas9), and 6 UAS-uCas9 lines with different uORF lengths ranging from 33 to 714 bp were generated (Port et al. 2020). In *Drosophila* wing imaginal discs, Cas9 immunostaining revealed a gradual decrease in Cas9 expression with increasing uORF length. Correspondingly, Cas9-induced apoptotic cell number gradually decreased, and target gene expression gradually increased as a result of lower Cas9 levels. Thus, this approach successfully tunes Cas9 expression levels, prevents Cas9-mediated cytotoxicity, and maintains Cas9 gene-editing efficiency in the wing imaginal disc (Port et al. 2020). We hypothesized that these uCas9 transgenes may present a suitable alternative to CRISPR-based mutagenesis in da neurons, and may be particularly useful for studies seeking to maintain the flexibility offered by the Gal4/UAS system.

In this study, we first validated Cas9-induced cytotoxicity in class IV da neurons using a transgene with no uORF. In da neurons, cytotoxicity has been previously identified by significant morphological defects in dendritic architecture (Poe et al. 2019). We also assessed Cas9-induced cytotoxicity/dendrotoxicity in 2 other widely studied da neuron types: class I and class III da neurons. We then characterized whether 3 extant publicly available uCas9 transgenes with varying uORFs could mitigate cytotoxicity in each cell type. These transgenes were uCas9(M), uCas9(L), and uCas9(VL) corresponding to medium, low, and very low expression of Cas9. We immunostained for Cas9 and found significant reductions in Cas9 protein levels with the uCas9(L) transgene in class I and class IV da neurons. Lastly, we confirmed gene editing efficiency using uCas9(L) in both class I and class IV neurons by (i) targeting GFP in da neurons expressing GFP-tagged proteins and (ii) producing loss-of-function dendrite phenotypes with previously validated gRNAs in class IV da neurons. Thus, we showcase a Gal4/UAS-compatible approach to minimizing Cas9-associated cytotoxicity via the use of available uCas9 transgenes and establish that these lowly expressed uCas9 transgenes are optimal for gene editing in *Drosophila* da neurons.

## Materials and methods

### Fly stocks and *Drosophila* culture

Class I da neurons were labeled by UAS-CD4::tdTomato under 2.21-Gal4 (Grueber et al. 2003a), class III da neurons were labeled by UAS-CD4::tdGFP under 19.12-Gal4 with glial repression under repo-Gal80 (Awasaki et al. 2008; Xiang et al. 2010), and class IV da neurons were labeled by ppk-CD4::tdGFP (Han et al. 2011). Cas9 transgenes used in this study include UAS-Cas9(H) (Bloomington #54595), UAS-uCas9(M) (Vienna #340002), UAS-uCas9(L) (Vienna #340003), and UAS-uCas9(VL) (Vienna #340004) (Port et al. 2020). To drive Cas9 expression in da neurons, 2.21-Gal4 was used for class I da neurons, 19.12-Gal4 was used for class III da neurons, and ppk-Gal4 (Grueber et al. 2007) was used for class IV da neurons. To visualize and drive expression of Cas9 in motor neuron terminals, we used nSyb-Gal4 from the Bloomington *Drosophila* Stock Center (51635). We used the following Bloomington *Drosophila* Stock Center gRNA lines to determine uCas9(L) gene editing efficiency: 67539 (QUAS.gRNA), 79393 (EGFP.gRNA), 81898 (Ptp69D.gRNA), and 81909 (Nsf1/2.gRNA). Animal genotypes for each figure can be found in Supplementary Table 1.

Adult fly crosses were used for 2 to 4 h synchronized egg lays and larvae were reared at ${\mathrm{22.5}}^{\circ }$ C in humidified incubators until 144 h after egg lay (AEL) (with the indicated exceptions). Identical culture conditions were used within datasets.

### Live imaging of *Drosophila* larvae

Larvae were briefly anesthetized using an isoflurane-soaked Kimwipe in a petri dish, then individually mounted on an agarose pad atop a glass slide. Dollops of vacuum grease were placed around the agarose pad, and a drop of glycerol was placed onto the larva. Coverslips were pressed onto the vacuum grease, with the larva oriented dorsal side up (or ventral side up for experiments imaging presynaptic terminals) and tape was used to secure the coverslip. Animals were imaged using a Zeiss LSM980, Zeiss LSM900, or Zeiss LSM700 microscope with a 20× air objective, or 40× water objective for experiments quantifying fluorescence intensity. For experiments reporting branch number and total dendrite length, Z-stacks containing the full dendrite arbor were acquired. For class IV dendrite arbors, the tiles function was used to ensure acquisition of the entire arbor.

### Fillet preparations and immunohistochemistry

Immunostaining for Cas9 levels was performed as described by Xu et al (Xu et al. 2024). Briefly, animals of each genotype were cultured for 144 h, fillet prepped ventral-side-up to maintain the integrity of dorsal da neurons, muscle was gently removed as in Tenenbaum and Gavis (Tenenbaum and Gavis 2016), and fillet preps were fixed with 4% PFA for 20 min on rocker set to 60 rpm at room temperature. Animals were then washed with 0.1% TritonX in DPBS^+/+^ (PBST), blocked with 5% normal goat serum in PBST, and treated with a mouse anti-Cas9 primary antibody (Novus Biologicals #7A9-3A3, 1:500) diluted in PBST overnight at 4 °C. After washing, samples were treated with goat anti-mouse Alexa Fluor 488 secondary antibodies (Invitrogen #A-10680, 1:750) for 2 h, washed again, then mounted using VectaShield Antifade Mounting Medium with DAPI (Vector Laboratories #H-1200-10).

### Quantification and statistical analyses

Image processing and analysis was done in Fiji/ImageJ. Z-stacks were maximum intensity projected and median filtered for clarity. Dendrite arbors were traced using the Simple Neurite Tracer plugin (Ferreira et al. 2014; Arshadi et al. 2021). Branch number and total dendrite length were acquired from Simple Neurite Tracer. To measure EB1::GFP intensity in da neuron cell bodies, the polygon selection tool was used to create an ROI around the cell body using the red channel representing CD4::tdTomato. Mean, median, and maximum intensity values within this ROI were then measured in the green channel representing EB1::GFP.

To quantify the internal coverage of class IV da neurons for loss-of-function experiments, we first processed maximum intensity projections using the following steps: Gaussian Blur = 0.8, Auto Local Threshold = Phansalkar (50), Analyze Particles = size(300-infinity) show (Masks). These images were then analyzed using the internal coverage macro with the following selections: grid size = 5 pixels, fill positive squares. To generate representative images with different fill colors, we slightly modified the Internal Coverage Macro code (see Supplementary Code for exact modification and a link to the original code). GraphPad Prism was used to generate graphs and perform statistical analyses. A significance threshold of *P* < 0.05 was used for all comparisons, and detailed information on specific statistical tests used can be found in figure captions.

## Results

### Cas9 expression is cytotoxic to da neurons

To assess Cas9-induced cytotoxicity in *Drosophila* da neurons, we imaged late third instar larvae expressing UAS-driven Cas9 in class IV, class I, or class III da neurons using the cell-type specific drivers ppk-Gal4, 2.21-Gal4, and 19.12-Gal4, respectively (Grueber et al. 2003a; Xiang et al. 2010). We call the expression of Cas9 without any uORF “Cas9(H)” for high expression. Expression of this Cas9(H) transgene caused severe defects in dendrite morphology in class IV ddaC neurons of larvae imaged at 144 h AEL (Fig. 1a). Quantifications of branch number and total dendrite length confirm that both measures are significantly reduced in Cas9(H)-expressing class IV ddaC neurons compared to wildtype (Fig. 1b). In agreement with previous literature, we describe this as cytotoxicity, even though we are not directly observing cell death. This corroborates previous findings of reduced branch density with Cas9(H) expression in ddaC neurons imaged in third instar animals (Poe et al. 2019). Next, we sought to determine whether Cas9(H)-induced dendrotoxicity in class IV da neurons is a developmental patterning defect or a progressive reflection of sustained toxic expression of Cas9. The class IV ppk-Gal4 driver begins expression in late embryogenesis (Grueber et al. 2003a), and Cas9-induced cytotoxicity was observed 144 h AEL, corresponding to roughly 130 h of Cas9(H) expression. To test whether there is an acute response to Cas9(H) expression, we assayed class IV da neuron morphology in larvae at earlier developmental stages, after roughly 36 h of Cas9(H) expression in 48 h AEL larvae (Fig. 1c). We found no genotype-specific differences in branch number or total dendrite length in larvae imaged at this earlier timepoint (Fig. 1d). Thus, the observed cytotoxicity in class IV ddaC neurons at 144 h AEL is likely a chronic response to sustained Cas9 expression, and not a developmental patterning defect.

**Fig. 1.**
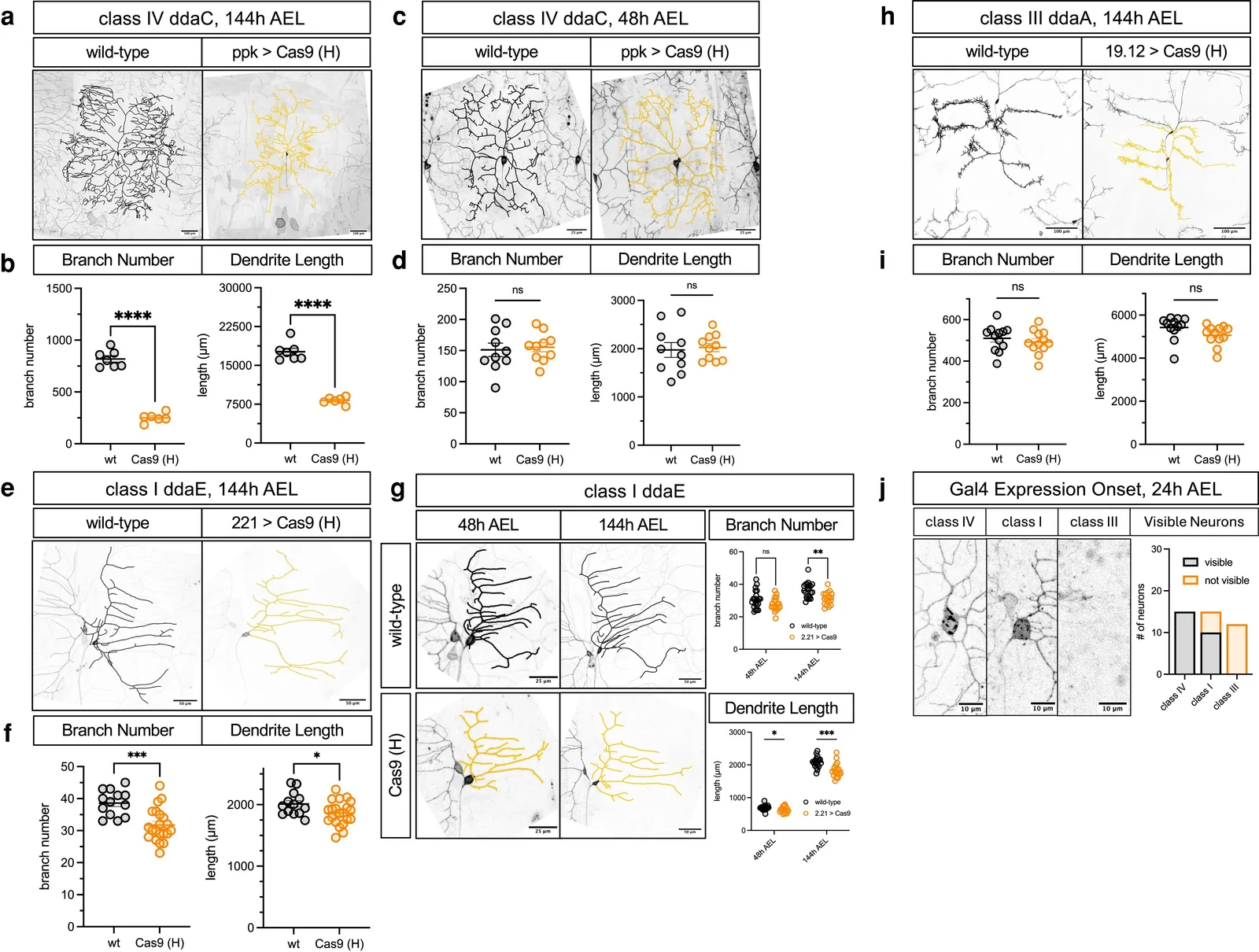
Expression of Cas9 in da neurons causes dendrotoxicity in a cell type-specific manner. a) Images of class IV ddaC neurons visualized with ppk-CD4::tdGFP in 144 h after egg lay (h AEL) wild-type or ppk > Cas9(H)-expressing neurons. UAS-Cas9.H (BDSC #54595) was used for all data in this figure. Traced dendrite arbors are overlaid with colors used for quantification in Fig. 1b. b) Quantifications of branch number and total dendrite length between wild-type and Cas9(H)-expressing ddaC neurons. Comparisons were made using unpaired t-test with Welch's correction. Average and error are mean ± SEM. **** *P* < 0.0001. N = 6 cells/group, 4 larvae/group, 1 to 2 cells/larvae. c) Images of class IV ddaC neurons visualized with ppk > CD4::tdTomato in 48 h AEL wild-type or ppk > Cas9(H)-expressing neurons. Traced dendrite arbors are overlaid with colors used for quantification in Fig. 1d. d) Quantifications of branch number and total dendrite length between wild-type and Cas9(H)-expressing ddaC neurons. Comparisons were made using unpaired t-test with Welch's correction. Average and error are mean ± SEM. *P* = 0.7476 for branch number and *P* = 0.7762 for dendrite length. N = 10 cells/group, 5 larvae/group, 2 cells/larvae. e) Images of class I ddaE neurons visualized with 2.21 > CD4::tdTomato in 144 h AEL wild-type or Cas9(H)-expressing neurons. f) Quantifications of branch number and total dendrite length between wild-type and Cas9(H)-expressing ddaE neurons. Comparisons were made using unpaired t-test with Welch's correction. Average and error are mean ± SEM. *** *P* < 0.001 for branch number and * *P* < 0.05 for total dendrite length. N = 13 to 21 cells/group, 4 to 6 larvae/group, 2 to 4 cells/larvae. g) Images of class I ddaE neurons repeatedly imaged at 48 h AEL and 144 h AEL. Quantification of branch number showed a trending difference between genotypes at 48 h AEL (*P* = 0.0696) but a significant difference at 144 h AEL. Quantification of total dendrite length showed significant differences between genotypes at both 48 h AEL and 144 h AEL. Comparisons were made using multiple unpaired t-test with Holm-Šídák correction. Average and error are mean ± SEM. ** *P* < 0.01 for branch number and * *P* < 0.05, *** *P* < 0.001 for total dendrite length. N = 24 cells/group, 8 larvae/group, 3 cells/larvae for the 48 h AEL timepoint. N = 18 to 24 cells/group, 6 to 8 larvae/group, 3 cells/larvae for the 144 h AEL timepoint. h) Images of class III ddaA neurons visualized with 19.12 > CD4::tdGFP, repo-Gal80 in 144 h AEL wild-type or Cas9(H)-expressing neurons. i) Quantifications of branch number and total dendrite length between wild-type and Cas9(H)-expressing ddaA neurons. Comparisons were made using unpaired t-test. Average and error are mean ± SEM. *P* = 0.464 for branch number and *P* = 0.093 for dendrite length. N = 12 cells/group, 6 larvae/group, 2 cells/larvae. j) Assessment of ppk-, 2.21-, and 19.12-Gal4 expression onset in 24 h AEL larvae. 15/15 imaged segments had visible class IV ddaC neurons, 10/15 imaged segments had visible class I ddaE neurons, and 0/15 imaged segments had visible class III ddaA neurons.

We extended this assessment to the more stable class I ddaE neurons (Fig. 1e), where we also observed a significant reduction in branch number and total dendrite length for those expressing Cas9(H) in 144 h AEL larvae (Fig. 1f). The class I 2.21-Gal4 driver also begins expression in late embryogenesis (Grueber et al. 2003a). In 48 h AEL larvae, Cas9(H)-expressing neurons showed a trending reduction in branch number (*P* = 0.07) and significant reduction in total dendrite length (Fig. 1g). When the identical neurons were re-imaged in 144 h AEL larvae, Cas9(H)-expressing neurons showed more significant reductions in both branch number and total dendrite length (Fig. 1g). These data show that even neurons with stable dendritic arbors are sensitive to the expression of Cas9(H), and that the onset of dendritic phenotypes resulting from high Cas9 expression may vary.

Lastly, expression of the Cas9(H) transgene in class III ddaA neurons did not affect either branch number or total dendrite length (Fig. 1h, i). The lack of cytotoxicity in class III da neurons may be due to the late expression onset of the 19.12-Gal4 driver relative to the ppk-Gal4 and 2.21-Gal4 drivers. To empirically test this, we imaged class IV, class I, and class III neurons at 24 h AEL in larvae expressing UAS-CD4::tdTomato (ppk-Gal4 and 2.21-Gal4) or UAS-CD4::tdGFP (19.12-Gal4). At 24 h AEL, class IV ddaC neurons were visible in 15/15 imaged segments, class I ddaE neurons were visible in 12/15 imaged segments, and class III ddaA neurons were not visible in any of the imaged segments (Fig. 1j). These results suggest the 19.12-Gal4 driver begins expression in class III ddaA neurons after the ppk/2.21-Gal4 drivers, which may account for the lack of Cas9(H) cytotoxicity in class III ddaA neurons.

We also assessed whether Cas9(H) may cause cytotoxicity to other cell types. We chose to examine this in the presynaptic terminals of motoneurons at the neuromuscular junctions (NMJs), as they are widely used to study the development, function, and plasticity of synapses. To visualize NMJs, we used nSyb-Gal4 and UAS-CD4::tdGFP, alongside the expression of Cas9(H). We chose to image terminals residing between muscles 6/7 on the ventral side of late third instar animals, as their location deeper into the animal allowed us to easily identify and distinguish these terminals from more superficial adjacent terminals. When driving Cas9(H) using nSyb-Gal4, these terminals looked similar to wild-type (Supplementary Fig. 1a). The presynaptic terminal of motoneurons expressing uCas9(L), designed to titrate Cas9 levels, also showed no gross changes in morphology (Supplementary Fig. 1a). Quantifications of large Type 1b and small Type 1s boutons revealed no significant changes in the bouton count between any of the genotypes tested (Supplementary Fig. 1b). These data reaffirm that Cas9(H) causes cytotoxicity only when paired with strong Gal4 drivers (Port et al. 2014).

### uCas9 transgenes are not cytotoxic to da neurons and titrate Cas9 levels

To titrate levels of Cas9 protein, we expressed 3 publicly available uCas9 transgenes with varying uORF lengths (uCas9(M), uCas9(L), and uCas9(VL), corresponding to medium, low, and very low Cas9 expression) or the normal Cas9(H) in class IV ddaC, class I ddaE, and class III ddaA neurons (Fig. 2a). For all 5 genotypes, in all 3 neuron types, we quantified the branch number and total dendrite length. In class IV ddaC neurons, Cas9(H) caused significant reductions in both branch number and total dendrite length compared to wild-type, but expression of uCas9(M), uCas9(L), or uCas9(VL) transgenes resulted in no differences to dendrite branch number or total dendrite length relative to wild-type (Fig. 2b, top row). Sholl analyses of traced class IV ddaC arbors show a drop in the number of intersections (dendrite crossings) as a function of distance from the soma for Cas9(H)-expressing neurons, but this drop in intersections is not observed in any of the uCas9-expressing neurons (Fig. 2c).

**Fig. 2.**
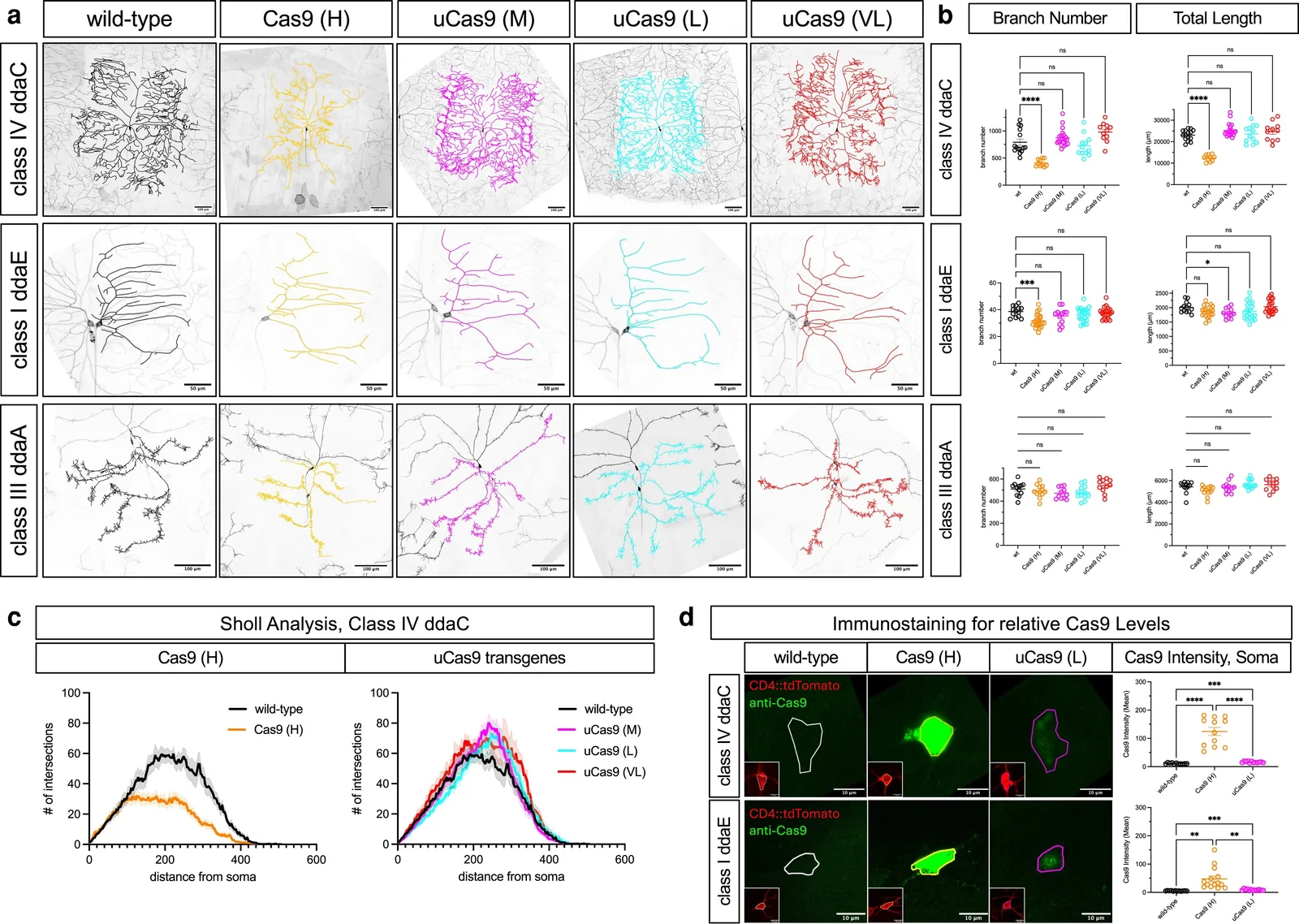
Expression of uCas9 transgenes does not cause reductions in branch number and results in lower Cas9 protein levels. a) Representative images of class IV ddaC (top row, visualized with ppk-CD4::tdGFP), class I ddaE (middle row, visualized with 2.21 > CD4::tdTomato), or class III ddaA (bottom row, visualized with 19.12 > CD4::tdGFP, repo > Gal80) neurons in third instar larvae as wild-type (first column, black overlay), Cas9(H)-expressing (second column, orange overlay, BDSC #54595), uCas9(M)-expressing (third column, magenta overlay, VDRC #340002), uCas9(L)-expressing (fourth column, cyan overlay, VDRC #340003), or uCas9(VL)-expressing (fifth column, purple overlay, VDRC #340004). b) Quantifications of branch number and total dendrite length between wild-type and Cas9-expressing neurons. Comparisons were made using Brown-Forsythe and Welch's ANOVA with Dunnett multiple comparisons correction. Average and error are mean ± SEM. * *P* < 0.05, ** *P* < 0.01, *** *P* < 0.001, **** *P* < 0.0001. N = 10 to 21 cells/group from 3 to 8 larvae for class IV ddaC neurons, 4 to 6 larvae for class I ddaE neurons, 4 to 6 larvae for class III ddaA neurons. c) Sholl analyses of ddaC neuron arbors with wild-type vs Cas9(H)-expressing ddaC neurons on the left and wild-type vs uCas9(M/L/VL)-expressing neurons on the right. Plots qualitatively depict a drop in intersection number for Cas9(H)-expressing neurons, but no drop in intersection number for neurons expressing various uCas9 transgenes. d) Immunostaining of Cas9 protein levels in wild-type, Cas9(H), and uCas9(L) larval fillet preps, with ppk-Gal4 (class IV ddaC) or 2.21-Gal4 (class I ddaE)-driven CD4::tdTomato. Quantification of Cas9 intensity in cell bodies showed significant differences between all groups. Brown-Forsythe and Welch ANOVA tests, with Dunnet multiple comparisons correction were used. Average and error are mean ± SEM. ** *P* < 0.01. *** *P* < 0.001. **** *P* < 0.0001. N = 11 to 14 cells/genotype, 3 larvae/genotype, 3 to 5 cells/larvae.

In class I ddaE neurons, the branch number is significantly reduced in Cas9(H)-expressing neurons, but normal in neurons expressing all of the uCas9 transgenes (Fig. 2b, middle row). Total dendrite length was unexpectedly slightly but significantly reduced in uCas9(M)-expressing class I ddaE neurons (Fig. 2b). Importantly, both uCas9(L) and uCas9(VL) neurons have normal dendrite length and branch number. In class III ddaA neurons, no changes in branch number or total dendrite length were observed with any of the transgenes tested, consistent with the later and lower cumulative level of expression from 19.12-Gal4, relative to the Gal4 drivers used for class IV and class I neurons (Fig. 2b). We chose to conduct further experiments using the uCas9(L) transgene as we detected no cytotoxic reductions in dendrite branch number or total dendrite length in either class IV ddaC or class I ddaE neurons with this transgene.

To determine whether uCas9 transgenes indeed lowered levels of Cas9 protein in da neurons, we immunostained 3rd instar larval fillet preps using anti-Cas9 antibodies in wild-type, Cas9(H)-expressing or uCas9(L)-expressing neurons. Quantification of somatic Cas9 levels revealed no Cas9 in wildtype neurons, high levels of Cas9 in Cas9(H)-expressing neurons, and drastic reductions of Cas9 expression in uCas9(L)-expressing class IV ddaC and class I ddaE neurons (Fig. 2d). This agrees with previous immunostaining data of Cas9 levels using these transgenes in the wing imaginal disc (Port et al. 2020), and provides evidence that the Cas9(H) transgene produces high levels of Cas9 protein, and that expression of the uCas9(L) transgene results in lower protein levels in da neurons.

Altogether, these data show that all 3 uCas9 variants fully rescue dendrite morphology in class IV ddaC neurons, but only uCas9(L) and uCas9(VL) have normal branch numbers and total branch lengths in class I neurons.

### The uCas9(L) transgene maintains gene editing efficiency in da neurons

Lastly, we wished to assess gene editing efficiency using the uCas9(L) transgene. To do this, we drove uCas9(L), the microtubule end-binding protein EB1::GFP, and CD4::tdTomato in class I ddaE neurons or class IV ddaC neurons, in either wild-type larvae or those ubiquitously expressing guideRNAs targeting GFP (Fig. 3a, b). Outlines of da neuron somas show regions used to quantify EB1::GFP intensity. In animals with GFP guideRNAs, the uCas9(L) transgene consistently reduced EB1::GFP intensity in both class I ddaE neurons (Fig. 3c) and class IV ddaC neurons (Fig. 3d).

**Fig. 3.**
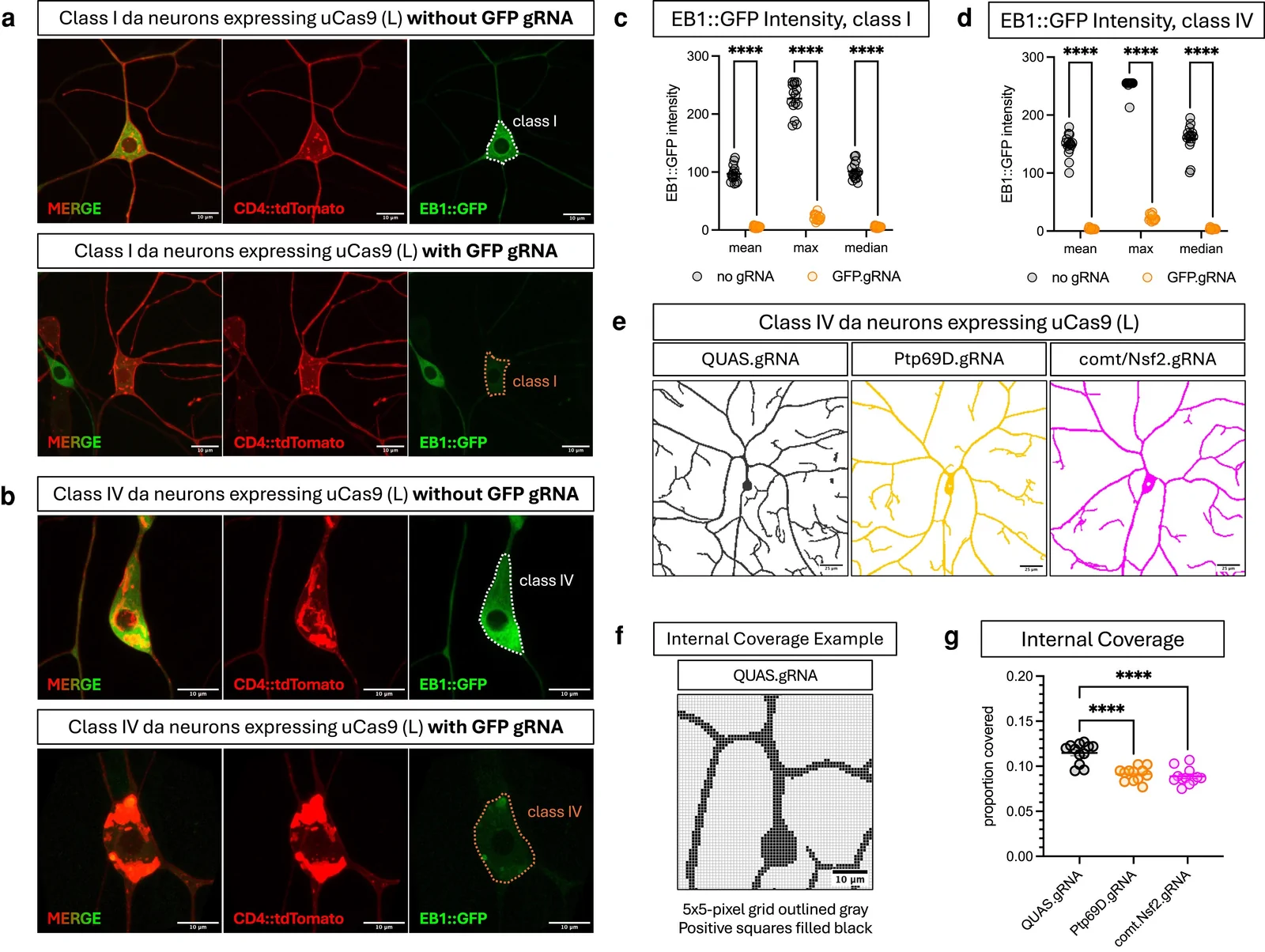
uCas9 transgenes are capable of gene editing and producing loss-of-function phenotypes in *Drosophila* da neurons. a) Class I da neurons expressing uCas9(L) without (top row) or with (bottom row) ubiquitously-expressed gRNAs targeting GFP. Class I ddaE cell bodies are outlined in the EB1::GFP channel for ease of interpretation. b) Class IV da neurons expressing uCas9(L) without (top row) or with (bottom row) ubiquitously-expressed gRNAs targeting GFP. Class IV ddaC cell bodies are outlined in the EB1::GFP channel for ease of interpretation. c) Quantification of EB1::GFP intensity in class I ddaE cell bodies. Multiple unpaired t-tests with Welch's Correction and multiple comparisons correction using Holm-Šídák were used to statistically test group differences. Average and error are mean ± SEM. **** *P* < 0.0001. N = 15 cells/group, 5 larvae/group, 3 cells/larvae. d) Quantification of EB1::GFP intensity in class IV ddaC cell bodies. Multiple unpaired t-tests with Welch's Correction and multiple comparisons correction using Holm-Šídák were used to statistically test group differences. N = 15 cells/group, 5 larvae/group, 3 cells/larvae. e) Class IV ddaC neurons expressing uCas9(L) with ubiquitously-expressed control QUAS.gRNA, Ptp69D.gRNA, or comt/Nsf2.gRNA demonstrating the ability of uCas9(L) to produce known dendritic loss-of-function phenotypes. Representative images are reflecting positively-filled squares after internal coverage analysis, described in the following panel. f) Zoomed in version (with ROIs displayed) of the QUAS.gRNA representative image in Fig. 3e depicting how the internal coverage macro quantifies area coverage. For these analyses, we chose a 5 × 5-pixel grid (grid ROIs outlined in light gray) and filled positive squares with their respective colors in Fig. 3g. g) Quantification of internal coverage showing the proportion of positively filled squares. Ordinary one-way ANOVA with Dunnet's multiple comparisons test was used to statistically test group differences from control (QUAS.gRNA). Average and error are mean ± SEM. **** *P* < 0.0001. N = 12 cells/group, 4 larvae/group, 3 cells/larvae.

To further validate gene editing efficiency, we sought to demonstrate the ability of uCas9(L) to produce loss-of-function phenotypes in class IV ddaC neurons with validated gRNA constructs targeting 2 genes known to be important for dendrite morphology: Ptp69D and comt/Nsf2 (dual gRNA). Prior work using genetic mutants or enhancer-driven Cas9 shows that Ptp69D or comt/Nsf2 loss of function results in significant reductions to dendrite density and total dendrite length (Poe et al. 2017; Poe et al. 2019). We found that class IV ddaC neurons expressing Cas9(L) with Ptp69D.gRNA or comt/Nsf2.gRNA exhibit simpler dendritic arbors (Fig. 3e). To quantify this, we measured the “internal coverage” of class IV ddaC neurons by overlaying a grid of user-specified size, then automatically counting the proportion of positively filled squares (Fig. 3f) (Sears and Broihier 2016). Using this approach, we found a significant reduction in the proportion of positive squares for neurons with Ptp69D.gRNA or comt/Nsf2.gRNA relative to QUAS.gRNA controls, reflecting a simplification of the dendrite arbor (Fig. 3g). Thus, uCas9(L) can effectively edit both GFP transgenes and endogenous gene targets in da neurons, and produce established loss-of-function phenotypes.

## Discussion

Overall, this study demonstrates a flexible approach with Gal4/UAS-dependent, Cas9-based mutagenesis in *Drosophila* sensory neurons, specifically highlighting the use of uCas9 transgenes designed to lower Cas9 protein levels and mitigate cytotoxicity. In addition to the cytotoxicity previously observed in highly dynamic class IV neurons, we extend the findings of cytotoxicity to the more stable class I neurons but find no evidence of cytotoxicity in class III neurons. Furthermore, our data from neurons imaged in young larvae suggest that the cytotoxic effects of Cas9 expression may be due to the cumulative expression of Cas9 over long periods of time at high levels. Acute vs chronic responses to Cas9 expression have been associated with DNA damage or immune cell responses, respectively, presenting an opportunity for future work interrogating the mechanisms of Cas9 cytotoxicity in da neurons (Ewaisha and Anderson 2023; Zhang et al. 2026).

After systematic analysis of various uCas9 transgenes, we identified uCas9(L) as the most optimal transgene for broad use in da neurons. We show that uCas9(L) expression drastically reduces Cas9 protein levels while maintaining mutagenesis efficiency and the ability to produce loss-of-function phenotypes. While we observed no Cas9-induced cytotoxicity in axon terminals of the NMJ, we only assessed the morphological consequences of nSyb-driven Cas9(H) expression. It is possible that other parameters of NMJ structure (ie terminals at different muscles) or function (ie synaptic transmission) may be altered with high Cas9 expression, and this study's Gal4/UAS-dependent strategy for titrating Cas9 levels may prove useful for future troubleshooting. Other strategies to mitigate Cas9 cytotoxicity in neurons via lowering of Cas9 protein levels have (i) used enhancer-driven Cas9 (ie ppk-Cas9 instead of ppk-Gal4 > UAS-Cas9) in *Drosophila* sensory neurons, (ii) shortened the half-life of Cas9 using geminin tags, which allows for endogenous regulation of Cas9 levels in rodent models, or (iii) expressed Cas9 proteins fused to degrons for drug-inducible, proteasomal degradation in human cells (Yang et al. 2018; Poe et al. 2019; Khajanchi et al. 2025). While effective, these approaches lose the flexibility offered by the widely used Gal4/UAS system in flies. Thus, the work presented here demonstrates a readily accessible means for CRISPR mutagenesis in *Drosophila* tissues, whose Cas9-associated confounds may go under-reported if cytotoxicity is encountered.

## Supplementary Material

jkag211_Supplementary_Data

## Data Availability

The authors affirm that all data necessary for confirming the conclusions of the article are present within the article, figures, and tables. Simple Neurite Tracer .traces files, representing traced reconstructions of dendrite arbors, have been submitted to and will be publicly available at NeuroMorpho.org, under the “Duarte and Thompson-Peer” archive. All fly stocks are available from Bloomington Drosophila Stock Center or the Vienna Drosophila Resource Center, with stock numbers specified in figure legends. Supplemental material available at G3 online.
