# Supplementary material for: Optimizing CRISPR/Cas9 mutagenesis in Drosophila dendritic arborization neurons to avoid cytotoxicity
Source: G3 (Bethesda). 2026 Sep 3;16(10):jkag211. doi: 10.1093/g3journal/jkag211 (PMC13643804; doi:10.1093/g3journal/jkag211)
Supplement: Supplementary file 1 [file jkag211_supplementary_data.pdf]

Supplementary Figure 1: nSyb-Gal4 driven Cas9 (H) or uCas9 (L) do not change gross morphology of presynaptic motor terminals.

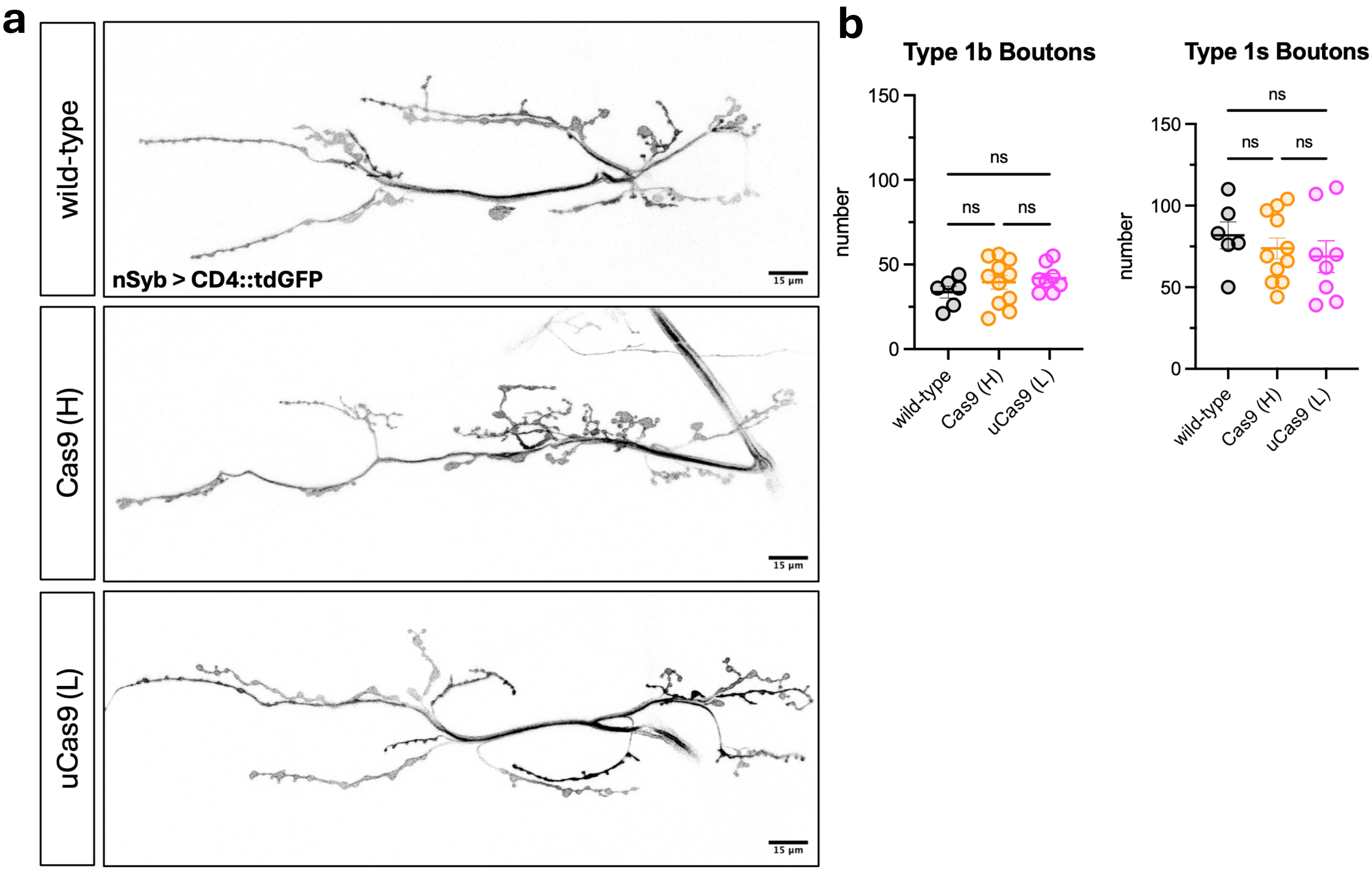

Supplementary Figure 1: nSyb-Gal4 driven Cas9 (H) or uCas9 (L) do not change gross morphology of presynaptic motor terminals. (a) Presynaptic terminals of muscles 6/7 in wild-type, Cas9 (H)-expressing, and uCas9 (L)-expressing motoneurons. **(b)** Quantification of Type 1b and Type 1s boutons show no change in bouton count between the listed genotypes. Ordinary one-way ANOVA with Tukey multiple comparisons correction were used to statistically test group differences. Average and error are mean ± SEM. N = 6-11 terminals/group, 2-4 larvae/group, 2-4 terminals/larvae.

Supplementary Table 1: Animal Genotypes

Figure	Panel	Label on Figure	Genotype
1	a	wild-type	; ppk-CD4::tdGFP / + ; ppk-gal4 / +
1	a	ppk > Cas9 (H)	; ppk-CD4::tdGFP / + ; ppk-Gal4 / UAS-Cas9
1	c	wild-type	; UAS-CD4::tdTomato / + ; ppk-Gal4 / +
1	c	ppk > Cas9 (H)	; UAS-CD4::tdTomato / + ; ppk-Gal4 / UAS-Cas9 (H)
1	e	wild-type	; ; 2.21-Gal4, UAS-CD4::tdTomato / +
1	e	2.21 > Cas9 (H)	; ; 2.21-Gal4, UAS-CD4::tdTomato / UAS-Cas9 (H)
1	g	wild-type, both ages	; ; 2.21-Gal4, UAS-CD4::tdTomato / +
1	g	Cas9 (H), both ages	; ; 2.21-Gal4, UAS-CD4::tdTomato / UAS-Cas9 (H)
1	h	wild-type	; ; 19.12-Gal4, UAS-CD4::tdGFP, repo-Gal80 / +
1	h	19.12 > Cas9 (H)	; ; 19.12-Gal4, UAS-CD4::tdGFP, repo-Gal80 / UAS-Cas9
1	j	class IV	; UAS-CD4::tdTomato / + ; ppk-Gal4 / +
1	j	class I	; ; 2.21-Gal4, UAS-CD4::tdTomato / +
1	j	class III	; ; 19.12-Gal4, UAS-CD4::tdGFP, repo-Gal80 / +
Supp 1	a-b	presynaptic terminals of the NMJ, wild-type	; UAS-CD4::tdGFP / + ; nSyb-Gal4 / +
Supp 1	a-b	presynaptic terminals of the NMJ, Cas9 (H)	; UAS-CD4::tdGFP / + ; nSyb-Gal4 / UAS-Cas9 (H)
Supp 1	a-b	presynaptic terminals of the NMJ, uCas9 (L)	; UAS-CD4::tdGFP / uCas9 (L) ; nSyb-Gal4 / +
2	a	class IV ddaC, wild-type	; ppk-CD4::tdGFP / + ; ppk-gal4 / +
2	a	class IV ddaC, Cas9 (H)	; ppk-CD4::tdGFP / + ; ppk-Gal4 / UAS-Cas9
2	a	class IV ddaC, uCas9 (M)	; ppk-CD4::tdGFP / UAS-uCas9 (M) ; ppk-gal4 / +
2	a	class IV ddaC, uCas9 (L)	; ppk-CD4::tdGFP / UAS-uCas9 (L) ; ppk-gal4 / +
2	a	class IV ddaC, uCas9 (VL)	; ppk-CD4::tdGFP / UAS-uCas9 (VL) ; ppk-gal4 / +
2	a	class I ddaE, wild-type	; ; 2.21-Gal4, UAS-CD4::tdTomato / +
2	a	class I ddaE, Cas9 (H)	; ; 2.21-Gal4, UAS-CD4::tdTomato / UAS-Cas9 (H)
2	a	class I ddaE, uCas9 (M)	; UAS-uCas9 (M) / + ; 2.21-Gal4, UAS-CD4::tdTomato / +
2	a	class I ddaE, uCas9 (L)	; UAS-uCas9 (L) / + ; 2.21-Gal4, UAS-CD4::tdTomato / +
2	a	class I ddaE, uCas9 (VL)	; UAS-uCas9 (VL) / + ; 2.21-Gal4, UAS-CD4::tdTomato / +
2	a	class III ddaA, wild-type	; ; 19.12-Gal4, UAS-CD4::tdGFP, repo-Gal80 / +
2	a	class III ddaA, Cas9 (H)	; ; 19.12-Gal4, UAS-CD4::tdGFP, repo-Gal80 / UAS-Cas9
2	a	class III ddaA, uCas9 (M)	; UAS-uCas9 (M) / + ; 19.12-Gal4, UAS-CD4::tdGFP, repo-Gal80 / +
2	a	class III ddaA, uCas9 (L)	; UAS-uCas9 (L) / + ; 19.12-Gal4, UAS-CD4::tdGFP, repo-Gal80 / +
2	a	class III ddaA, uCas9 (VL)	; UAS-uCas9 (VL) / + ; 19.12-Gal4, UAS-CD4::tdGFP, repo-Gal80 / +
2	d	class IV ddaC, wild-type	; UAS-CD4::tdTomato / + ; ppk-Gal4 / +
2	d	class IV ddaC, Cas9 (H)	; UAS-CD4::tdTomato / + ; ppk-Gal4 / UAS-Cas9
2	d	class IV ddaC, uCas9 (L)	; UAS-CD4::tdTomato / UAS-uCas9 (L) ; ppk-Gal4 / +
2	d	class I ddaE, wild-type	; ; 2.21-Gal4, UAS-CD4::tdTomato / +
2	d	class I ddaE, Cas9 (H)	; ; 2.21-Gal4, UAS-CD4::tdTomato / UAS-Cas9 (H)
2	d	class I ddaE, uCas9 (L)	; UAS-uCas9 (L) / + ; 2.21-Gal4, UAS-CD4::tdTomato / +
3	a	Class I da neurons expressing uCas9 (L) without GFP gRNA	; UAS-uCas9 (L) / UAS-EB1::GFP ; 2.21-Gal4, UAS-CD4::tdTomato / +

3	a	Class I da neurons expressing uCas9 (L) with GFP gRNA	; UAS-uCas9 (L) / UAS-EB1::GFP ; 2.21-Gal4, UAS-CD4::tdTomato / U6:3-GFP.gRNA
3	b	Class IV da neurons expressing uCas9 (L) without GFP gRNA	; UAS-uCas9 (L) / UAS-EB1::GFP ; ppk-Gal4, UAS-CD4::tdTomato / +
3	b	Class IV da neurons expressing uCas9 (L) with GFP gRNA	; UAS-uCas9 (L) / UAS-EB1::GFP ; ppk-Gal4, UAS-CD4::tdTomato / U6:3-GFP.gRNA
3	e/g	QUAS.gRNA	; UAS-uCas9 (L) / U6-QUAS.gRNA ; ppk-Gal4, UAS-CD4::tdTomato / +
3	e/g	Ptp69D.gRNA	; UAS-uCas9 (L) / + ; ppk-Gal4, UAS-CD4::tdTomato / U6-Ptp69D.gRNA
3	e/g	comt/Nsf2.gRNA	; UAS-uCas9 (L) / + ; ppk-Gal4, UAS-CD4::tdTomato / U6:3-comt/Nsf2.gRNA

Supplementary Code for Internal Coverage Analyses :

Original code can be found here.

Changes to original code are **bolded and colored red** in code block below.

```
n = roiManager("count");
for(i=1;i<n;i++){
	roiManager("select", i);
	getRawStatistics(area, mean, min, max, std, histogram);
		if (max>enterthreshold)
		{
			PositiveROI +=1;
			if (fillpositiveboxes==1)
			{
				Roi.setFillColor("magenta");
				run("Add Selection...");
}
}
}
```
